# Is There a Relation between ABO Blood Groups and Clinical Outcome in Patients with Pemphigoid? A Case-Control Study

**DOI:** 10.1155/2016/3916750

**Published:** 2016-06-29

**Authors:** Sedigheh Bakhtiari, Parviz Toosi, Somayyeh Azimi, Nafiseh Esmaili, Ali Montazami, Nasrin Rafieian

**Affiliations:** ^1^Oral Medicine Department, Dental School, Shahid Beheshti University of Medical Sciences, Tehran, Iran; ^2^Skin Research Center, Tehran University of Medical Sciences, Tehran, Iran; ^3^The International Research Collaborative - Oral Health and Equity, School of Anatomy, Physiology and Human Biology, University of Western Australia, Crawley, WA 6009, Australia; ^4^Skin Research Center, Shahid Beheshti University of Medical Sciences, Tehran, Iran; ^5^Shahid Beheshti University of Medical Sciences, Tehran, Iran; ^6^Department of Oral Medicine, Faculty of Dentistry, Alborz University of Medical Sciences, Karaj, Iran

## Abstract

*Background.* Relationship between blood groups and dermatologic diseases remains controversial and was not yet fully elucidated nor explained clearly. The aim of this study was to examine if any relation exists between different types of pemphigoid diseases and ABO blood group.* Methods.* In this case-control study, 159 pemphigoid patients and 152 healthy matched-controls were evaluated. All blood group (including Rh status) data for the study was obtained from the hospital medical records. Statistical comparisons were completed with chi-square test and logistic regression.* Results.* Blood group “O” was found in 32.9% of patients and 38.2% of control group. Blood group “A” was found among 30.8% of patients and 34.2% of control group, while group “B” was reported in 27.4% of cases and 21.1% of controls and “AB” was identified among 8.9% of patients and 6.6% of control group. 84.9% of patients were Rh positive, while in the control group 86.2% of patients were Rh positive. No significant differences were found regarding ABO blood groups (*P* = 0.46) or Rh (*P* = 0.76) between pemphigoid patients and control group. Also, older females had the higher risk of developing bullous pemphigoid.* Conclusion.* We found no relationship between ABO blood groups and pemphigoid disease.

## 1. Introduction

Pemphigoid is a subepithelial bullous disease that is characterized by an autoimmune reaction that weakens the basement membrane. There are 3 types of pemphigoid. Bullous pemphigoid (BP) is the most common subepithelial blistering disease, which is mainly observed in adults over 60 years old [1]. Mucous membrane pemphigoid (MMP), previously called cicatricial pemphigoid (CP), is a subtype of disease that primarily affects mucous membrane of patients over 50 years old and causes mucosal ulceration and scars [1–3]. Another type of pemphigoid is pemphigoid gestationis (PG) that refers to pregnancy. The cutaneous hallmark of pemphigoid is tense blisters that occur on the arms, legs, abdomen, and mucous membranes. Oral lesions are also observed in pemphigoid diseases that are similar to pemphigus, which are smaller and less painful and extensive labial involvement is not observed. The disease is sometimes self-limiting but treatment is necessary [2, 3].

Blood groups as a hematological marker have been assessed in several studies. Genetic factors such as blood group antigens may affect the risk, severity, and development of some medical conditions. The association between blood groups and different diseases such as various malignancies, peptic ulcer, infection, diabetes mellitus, dermatologic disease, heart disease, dental caries, and infectious disease had been studied with different results [4–7]. Some studies have shown increased related risks of some diseases. For example, the association between ABO groups with several malignancies, hypercholesterolemia, thrombosis, myocardial infarction, duodenal ulcer, infections, and autoimmune diseases is reported [8]. Also, Mortazavi et al. [9] found that squamous cell carcinoma (SCC) has been more common in blood group B. Moreover, case-control studies have reproducibly showed significant associations of ABO blood group and particular HLA antigens with various human diseases. These were mainly autoimmune disorders such as pemphigus vulgaris, type 1 diabetes, multiple sclerosis, rheumatoid arthritis, psoriasis, and celiac disease [10]. Some studies evaluated the relation between blood group and pemphigus disease [8, 10–15]; for instance, Valikhani et al. [11] could not find any relation between blood groups ABO and Rh factor with pemphigus disease among the Iranian population. Also Tirado-Sánchez and Ponce-Olivera [15] concluded the nonexistent relationship between ABO and Rhesus blood groups and pemphigus vulgaris. To the best of our knowledge, we could not find any study to evaluate the association between blood groups and pemphigoid. Since distribution of ABO genes is different among ethnic and social groups, the aim of this study was to determine any linkage between ABO and pemphigoid in the Iranian population.

## 2. Materials and Methods

In this case-control study, all patients with a history of pemphigoid that were referred to Razi Hospital Tehran (Iran) from 2005 to 2013 were evaluated. Razi Hospital is one of the leading dermatologic clinical centers in Iran and patients come from a wide area of Iran.

The data which included demographic data (age and gender), the site of pemphigoid involvement (cutaneous lesion, oral mucosa, or both), pemphigoid type (BP, MMP, and PG), and blood groups (A, B, AB, and O) as well as Rh (positive or negative) were extracted from the patients' records. The patients without blood group and Rh history in their records were excluded from the study. The final number of patients in the data frameset was 159. Totally, 152 control patients were randomly chosen from healthy people who were referred to Razi Hospital, and their blood groups as well as Rh were collected from their medical records.

This study has been independently reviewed and approved by ethics committee of Shahid Beheshti University of Medical Sciences; also, this research has been conducted in full accordance with the world medical association Declaration of Helsinki. We confirm that patients' information remained confidential and data was anonymized and deidentified prior to analysis.

The data were analyzed with SPSS (Statistical package for Social Sciences 19; SPSS 19, Chicago, IL, USA). The frequency of blood groups and Rh of pemphigoid patients as well as control group were determined. The chi-square test was used to compare qualitative variables, and the logistic regression test was used to examine the relationship between blood groups and Rh in pemphigoid patients and control group. *P* value of < 0.05 was considered statistically significant.

## 3. Results

In pemphigoid patients, 7 cases (2.3%) had MMP, 146 cases (46.9%) had BP, and 6 cases (1.9%) had PG (Table 1).

Over 14% of MMP patients had only oral involvement and the coincidence of oral and cutaneous lesions was seen in 71.4% of patients while in BP group there was no case with only oral involvement. However, cutaneous involvement without oral lesions was common in BP patients. There was no oral involvement in cases with PG (Table 2).

Distribution of blood group and Rh in different types of pemphigoid and control group is shown in Table 3. However, because of the limited number of MMP and PG patients, statistical comparisons were only completed with BP cases. Blood group O was found in nearly a third of all BP patients (*n* = 48, 32.9%) and controls (*n* = 58, 38.2%). Similarly, about a third of cases (*n* = 45, 30.8%) and controls (*n* = 52, 34.2%) had blood group A. There was no significant difference between various blood groups in patients with BP and the control group (*P* = 0.46). Also, 124 (84.9%) patients were Rh positive and 22 (15.1%) were Rh negative. In the control group, 131 patients (86.2%) were Rh positive and 21 cases (13.8%) were Rh negative. Chi-square test results showed no significant differences in the distribution of Rh between BP and control group (*P* = 0.76). In addition, logistic regression demonstrated that older females significantly had higher risk of developing BP disease (*P* = 0.018 and 0.0001, resp.); in other words, the odds of having disease in females are twice compared to males. Increasing a year of age will increase the chance of disease by 6%. However, there was not any significant relationship among different blood groups (Table 4). We could not do statistical examination between cutaneous and oral involvement due to limited number of patients that have only oral involvement.

## 4. Discussion

This study for the first time examined the relationship between pemphigoid diseases and ABO and Rh blood groups in 159 patients with various types of pemphigoid and 152 healthy controls in Iranian population. Statistical analyses did not show any significant relationship. This study collected 146 cases of BP, 7 cases of MMP, and 6 cases of PG and studied genders, ages of disease onset, and involvement of either skin or oral mucosa. There are some studies that evaluate the relationship of various blood groups in a variety of diseases [9, 11–22], such as pemphigus vulgaris [11–15] and oral cancer [9, 16–18].

In 1969, Altobella [23] assessed the relation between blood groups and various dermatoses and concluded that 60% of pemphigus vulgaris patients had blood group O; however, no comparison was done with the control group. Also, the result of Grob and Inderbitzin study [17] indicated that vulgaris pemphigus is more frequent among people with blood group O. Shahkar et al. [14] investigated blood groups of 56 Iranian patients suffering from pemphigus. According to the results of recent surveys, no significant relation has been observed between blood groups ABO and vulgaris pemphigus. Valikhani et al. [11] did not find any significant relationship between blood groups ABO or blood factor Rh with subgroups of pemphigus in Iranian population. Also, Tirado-Sánchez and Ponce-Olivera [15] concluded that there is no relation between blood groups ABO and Rh among pemphigus patients and healthy people.

Frequency of blood groups in various groups of cancer patients in northern India [16] showed that, compared with the control group, people with blood group A had a higher rate of SCC; and Mortazavi et al. [9] reported that SCC was more prevalent among people with blood group B, and it was less prevalent among those with blood group AB than among healthy people.

In the present study, most of patients, as well as the control group, had blood group O (32.9% and 38.2%, resp.). In line with our study, Mortazavi et al. [9] reported that blood group O is the most common blood group in Iranian population. Based on the present survey, 84.9% of the patients had Rh+ and 15.4% Rh−. In the control group, 86.2% had Rh+ and 13.8% Rh−. No significant difference was noticed in pemphigoid patients and the control group in distribution of Rh. No previous research has ever evaluated the relation between positive and negative Rh with pemphigoid.

Moreover, there are some reports about associations between susceptibility to certain diseases and Lewis antigen and secretor status phenotype based on linkage among ABH, Lewis blood group system, and secretor status. For instance, in 2014, Shahidi-Dadras and Golfeshan [24] evaluated Lewis blood group antigens and secretor status in pemphigus vulgaris. They showed that Le/b-negative nonsecretor individuals are more susceptible to pemphigus vulgaris. Also, in 2004, Xuan et al. [25] evaluated the association of Lewis blood group expression with ocular cicatricial pemphigoid (OCP) and reported that the anti-Le (a) and anti-Le (b) immunoreactivity of epithelial and goblet cells was considerably decreased, and more than 40% of OCP patients had the nonsecretor phenotype, which was significantly higher than the healthy population. They concluded that OCP might be associated with a nonsecretor phenotype. However, in the present study we did not evaluate OCP phenotype; MMP has several clinical variants or manifestations, and this study only included oral, cutaneous, and mixed type. This is a preliminary study for pemphigoid disease and evaluating Lewis antigens and nonsecretor phenotype in diffident types of pemphigoid disease is recommended for future studies.

## 5. Conclusion

From the data of this study, it can be concluded that there was no clear relation between blood group ABO and Rh group with type of pemphigoid disease. Considering the fact that there is a conflict among results of studies, a systematic review to find a clear relationship between ABO blood groups and autoimmune disease is recommended for future studies.

## Figures and Tables

**Table 1:** Demographic characteristic of participants.

Type	Age	Male (%)	Female (%)	Total (%)
Mucous membrane pemphigoid	57.6 ± 13.9	5 (71.4%)	2 (28.6%)	7 (100%)
Bullous pemphigoid	68.3 ± 17.9	66 (45.2%)	80 (54.8%)	146 (100%)
Pemphigoid gestationis	31 ± 6.7	0	6 (100%)	6 (100%)
Control group	54.1 ± 13.4	86 (56.6%)	66 (43.4%)	152 (100%)

**Table 2:** Distribution of patients based on the site of involvement.

Group	Oral lesion	Cutaneous lesion	Oral & cutaneous lesions	Total
Mucous membrane pemphigoid	1 (14.3%)	1 (14.3%)	5 (71.4%)	7 (100%)
Bullous pemphigoid	0	109 (74.7%)	37 (25.3%)	146 (100%)
Pemphigoid gestationis	0	6 (100%)	0	6 (100%)

**Table 3:** Distribution of blood group and Rh in different pemphigoid types and control group.

Blood group	A		B		O		AB		Rh+		Rh−	
	Number	%	Number	%	Number	%	Number	%	Number	%	Number	%
Mucous membrane pemphigoid	3	42.9	2	28.6	2	28.6	0	0.0	5	71.4	2	28.6
Bullous pemphigoid	45	30.8	40	27.4	48	32.9	13	8.9	124	84.9	22	15.1
Pemphigoid gestationis	3	50.0	2	33.3	1	16.7	0	0.0	5	83.3	1	16.7
Control group	52	34.2	32	21.1	58	38.2	10	6.6	131	86.2	21	13.8

**Table 4:** Relationship between blood groups and Rh in pemphigoid patients with regard to age and sex (logistic regression).

Variables	*B*	SE	*P* value	OR	CI	
					Lower	Upper
Sex (male)	−0.623	0.0262	0.018	0.0536	0.0321	0.89
Age	0.0059	0.0009	0.0001	1.061	1.043	1.07
Blood group O^*∗*^			0.0484			
Blood group A	0.0218	0.0312	0.0485	1.244	0.0674	2.29
Blood group B	0.0507	0.0342	0.0139	1.660	0.0848	3.24
Blood group AB	0.0427	0.0537	0.0380	1.603	0.0559	4.59
RH+	−0.0235	0.0390	0.0547	0.0795	0.0368	1.69
Constant	−3.374	0.0669	0.000	0.0034		

*B*: estimate.

SE: standard error.

OR: odds ratio, EXP(*B*).

CI: confidence interval for odds ratio.

*∗*: reference blood group.
